# Reverse Takotsubo Cardiomyopathy in a Critically Ill Patient in the ICU: A Case Report With Literature Review

**DOI:** 10.7759/cureus.35752

**Published:** 2023-03-04

**Authors:** Muhammad Ghallab, Ibrahim Mohamed, Muhammad Haseeb ul Rasool, Salma Abdelmoteleb, Allison Foster, Zakaria Alagha, Ashraf Sliem, Md Ripon Ahammed, Nicole C Noff, Daniel Miller, Giovina Collura

**Affiliations:** 1 Internal Medicine, Icahn School of Medicine at Mount Sinai, NYC Health + Hospitals/Queens, New York City, USA; 2 Internal Medicine, Cairo University School of Medicine, Giza, EGY; 3 Medicine, Icahn School of Medicine at Mount Sinai, NYC Health + Hospitals/Queens, New York City, USA; 4 Internal Medicine, Marshall University Joan C. Edwards School of Medicine, Huntington, USA; 5 Internal Medicine, Flushing Hospital Medical Center, New York City, USA; 6 Cardiology, Icahn School of Medicine at Mount Sinai, NYC Health + Hospitals/Queens, New York City, USA

**Keywords:** stress induced cardiomyopathy, acute coronary syndrome, reverse takutsubo cardiomyopahty, takutsubo cardiomyopathy, stress cardiomyopathy

## Abstract

Takotsubo, or stress cardiomyopathy (SC), is described as a transient systolic dysfunction of the apical segments of the left ventricle mainly triggered by emotional or physical stress resembling the presentation of an acute coronary syndrome in the absence of obstructive coronary artery disease. Reverse Takotsubo SC is a rare variant of SC that presents with basal ballooning instead of apical ballooning seen in classic SC. We present a case of a 74-year-old male who was admitted to the ICU with septic shock. Laboratory test results showed elevated troponin. An echocardiogram showed reduced cardiac contractility and relative hypokinesis of the basal and mid segments compared to the apical segments, consistent with reverse Takotsubo SC, which recovered after 10 days. It can happen in critically ill patients in the ICU secondary to severe sepsis and could contribute to hemodynamic worsening, affecting the final clinical outcomes.

## Introduction

The "broken heart syndrome," also known as Takotsubo cardiomyopathy (TTC), stress cardiomyopathy (SC), or TTC, was initially identified in Japanese women in 1983 [1]. It is defined as an abrupt but frequently reversible systolic malfunction of the left ventricle's apical portions with a clinical presentation often approaching acute coronary syndrome (ACS) but without evidence of obstructive coronary artery disease on angiography [2]. Stress, either emotional or physical, is the leading cause of the syndrome [2]. Most TTC patients regain cardiac function within a few days or weeks [2]. Transient cardiac apical akinesis or hypokinesis and basal hyperkinesis accompanied by apical ballooning are the hallmarks of TTC [2]. TTC was given its name because of the heart's distinctive apical ballooning, which gave it a shape resembling a "Takotsubo," a Japanese pot used to catch octopuses [3]. Several TTC variants have been identified based on the locations of the ventricular wall motion abnormalities, such as the midventricular and basal walls [3]. Reverse Takotsubo cardiomyopathy (rTTC) is a unique form of TTC in which the basal and midventricular segments of the left ventricle are akinetic or hypokinetic associated with apical hyperkinesis and present with basal ballooning rather than apical ballooning, as seen in classic TTC [3]. According to estimates, 2% of all patients who test positive for troponin and have suspected ACS will develop TTC [4,5]. In the published literature, the percentage of patients presenting with the rTTC variation out of all TTC patients has varied, ranging from 1% to 23% [6,7]. We present a case of sepsis-induced rTTC.

## Case presentation

**Figure 1:**
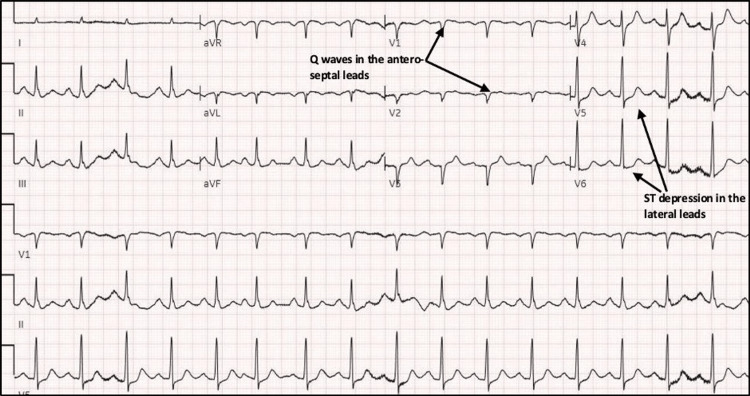
12 leads electrocardiogram shows sinus tachycardia, left atrial enlargement, upsloping ST segment depression in the V4-V6 leads, and Q waves in V1-V3 leads.

A 74-year-old male with a past medical history significant for chronic obstructive pulmonary disease (COPD) was brought into the ED by the EMS for shortness of breath. The patient was found to be obtunded and agitated. He was intubated in the ED because of severe hypoxia and respiratory distress and was started on norepinephrine for severe hypotension. The patient was transferred to the ICU for ventilatory support on continuous propofol and fentanyl drip. The patient's EKG (Figure 1) showed sinus rhythm with left atrial enlargement and upsloping ST segment depression in the lateral leads and Q waves in the anteroseptal leads. 

The patient's laboratory results (Table 1) were significant for leucocytosis, severe respiratory acidosis markedly elevated D-dimer, and elevated troponin. 

**Table 1:** Summary of the patient's initial test results. ALT: Alanine transaminase; AST: Aspartate transaminase.

Labs	Value	Reference range
Complete blood count		
Hemoglobin (Hb)	14.6 g/dl	12.0-16.0 g/dL
WBC	13.76 x 10(3)/mcL	4.8-10.8 x 10(3)/mcL
Platelets	195 x 10(3)/mcL	150-450 x 10(3)/mcL
Kidney functions tests		
Blood urea nitrogen	34 mg/dL	6-23 mg/dL
Creatinine	1.2 mg/dL	0.5-1.2 mg/dL
Sodium	136 mmol/L	136-145 mmol/L
Potassium	4.8 mmol/L	3.5-5.1 mmol/L
Liver function tests		
ALT	19 U/L	0-33 U/L
AST	39 U/L	5-32 U/L
Coagulation profile		
D-dimer	4,068 ng/mL	≤285 ng/mL
Activated partial thromboplastin time (aPTT)	30.0 seconds	25.1-36.5 seconds
Prothrombin time (PT)	14.6 seconds	10.0-13.0 seconds
Arterial blood gases (ABG)		
PH	7.04	7.35-7.45
PO2	163 mmHg	83-108 mmHg
PCO2	81 mmHg	32-35 mmHg
Lactate	3.4 mmol/L	0.4-0.8 mmol/L
Troponin-I (1)	0.853 ng/ml	≤0.010 ng.ml
Troponin-I (2)	1.260 ng/ml	≤0.010 ng.ml
Troponin-I (3)	0.784 ng/ml	≤0.010 ng.ml

**Figure 2:**
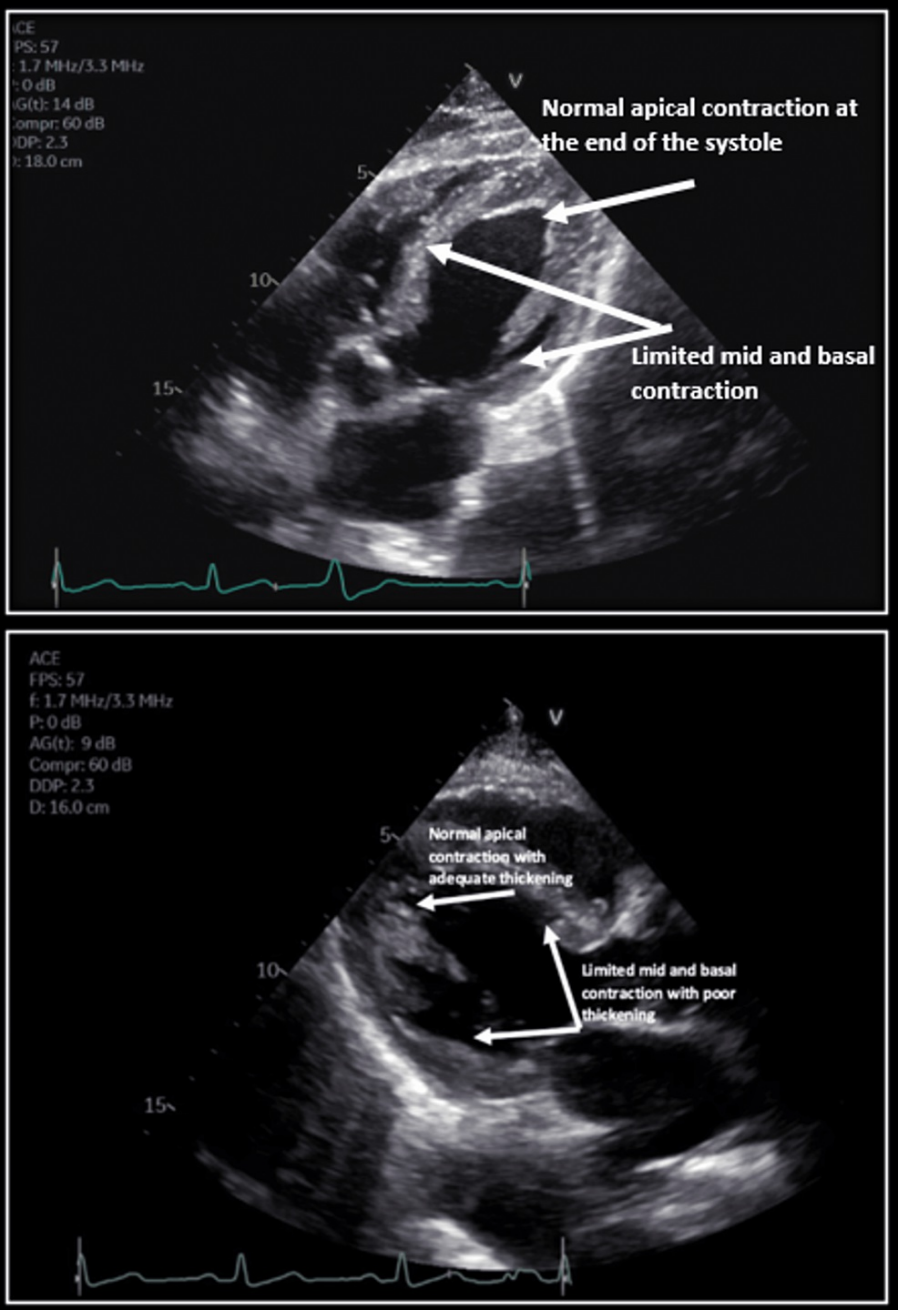
Transthoracic echocardiogram, apical-four chamber view (upper photo), parasternal long axis view (lower photo) at the end of the systole showing fully contracting apex and limited basal and mid segments contraction.

The patient was started on methylprednisolone and ipratropium bromide/albuterol nebulization to treat COPD exacerbation. Transthoracic echocardiogram (TTE) was performed (Figure 2).

**Figure 3:**
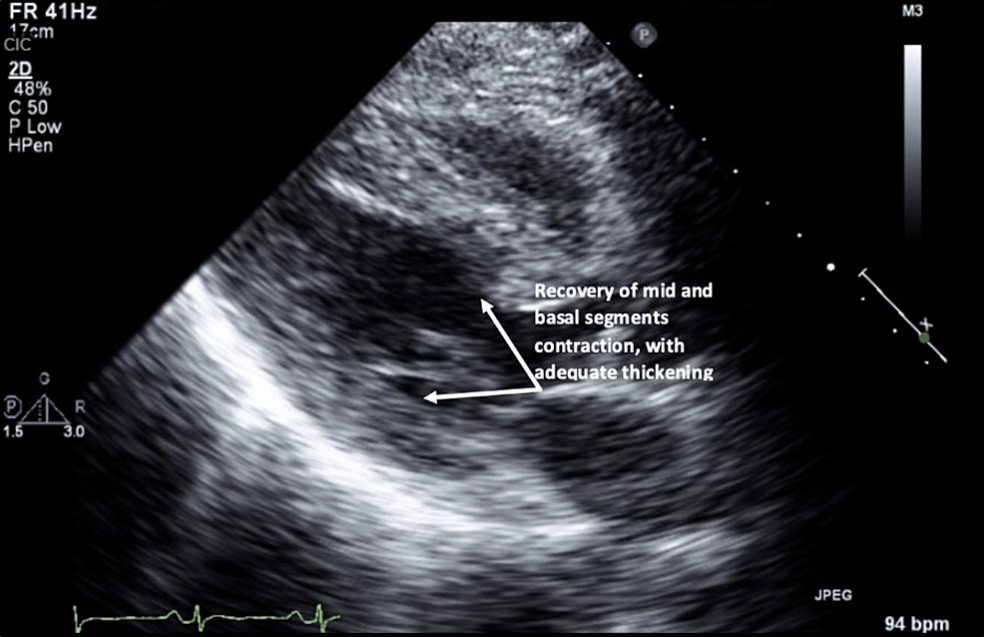
Transthoracic echocardiogram, parasternal long axis view at the end of the systole showing fully contracting apex along with recovery of the basal and mid segments contractile function.

It was significant for moderately decreased LV ejection fraction (LVEF) of 35-40%, grade II (moderate) left ventricular diastolic dysfunction, moderate mitral regurgitation, relative hypokinesis of the basal and mid segments compared to the apical segments, and consistent with reverse Takotsubo's cardiomyopathy. The possibility of non-ST-elevation myocardial infarction (NSTEMI) could not be ruled out in the presence of EKG changes and elevated troponin, so the patient received loading doses of aspirin and ticagrelor in addition to high-intensity statins and therapeutic anticoagulation with enoxaparin. Computed tomography pulmonary angiography was performed, which showed no evidence of pulmonary embolism. The enoxaparin was stopped on day two of admission, while the rest of the treatment was continued. Repeated trials to wean the patient off the ventilator had failed during this period due to increased work of breathing upon de-escalation of therapy. A coronary angiogram could not be done because of the patient's hemodynamic instability and non-improved conscious level with suspected significant brain damage. An echocardiogram (Figure 3) on day 10 of admission was done.

It showed improvement of LVEF (65-70), with the apex now relatively hyperkinetic compared to the base, consistent with the resolution of previously documented cardiomyopathy. Despite improvement in the LVEF and cardiomyopathy, the patient still required pressors due to intermittent hypotension. The patient continuously stayed on ventilatory support, with no purposeful respiratory or cerebral function gain. At that stage of the disease, the patient was progressively de-escalated to comfort measures per the family's request. The patient had palliative extubation and was declared dead on day 75 of admission.

## Discussion

Catecholamine-mediated damage is assumed to be the primary cause of TTC, typically observed in critically ill patients or physical or mental stress [8]. The increased release of catecholamines from pheochromocytomas and paragangliomas, as well as the intravenous administration of catecholamines and other receptor agonists, as well as other hypercatecholaminemic states seen in acute cerebral disorders like subarachnoid hemorrhage and severe head injury, have all been found to be associated with rTTC [9-12]. Dysregulated corticosteroid hormonal balance leads to a maladaptive catecholaminergic response in cardiac tissue and is yet another important determinant of TTC. Hence, both excess and deficiency of corticosteroids can lead to TTC and rTTC [13].

In animal models, the development of acute reversible apical hypokinesia of the heart following intravenous catecholamine administration has been demonstrated in vivo experiments. The same outcomes have been found when rats are exposed to emotional stresses like immobility [14].

A retrospective study was conducted in Japan in 328 patients with TTC variants and subarachnoid hemorrhage. The results showed that plasma epinephrine levels were significantly higher in rTTC patients than in TTC patients, while plasma norepinephrine levels did not differ significantly between the two groups [10]. Patients with TTC who had endomyocardial biopsy may have suffered cardiomyocyte damage due to elevated catecholamine levels. This is because myofibrillar degeneration, contraction band necrosis, and mononuclear leukocyte infiltration are histologic signs of myocyte injury linked to catecholamine toxicity [14] in individuals with TTC.

It is unknown how exactly catecholamines can cause its toxic effect. However, the proposed mechanism is that high doses of epinephrine cause direct toxicity to cells, leading to an increase in cyclic AMP and calcium levels, which in turn cause the formation of free oxygen radicals, which in turn cause the expression of stress response genes and induce apoptosis [15,16]. Catecholamines affect adrenoreceptors, which peak in density near the base of the heart during adolescence and progressively move toward the apex as we age. This explains why older women experience traditional TTC while younger patients experience rTTC. These changes may be brought about by regional variations in adrenoreceptor sensitivity or individual differences in myocardial innervation in addition to adrenoreceptor density [17]. According to a study of 60 patients, patients with rTTC present at a younger age, with a mean age of 36, compared to 62 for other forms [18].

Angiograms of patients with TTC have revealed multifocal coronary vasospasm that causes apical ballooning [19]. A case report that revealed reversible ST-segment elevation with intra-coronary nitroglycerin in a patient with TTC has also shown catecholamine-induced vascular spasm [20-21]. Due to aberrant LV wall motion in the vast regions of the myocardium, which is dynamic rather than stationary, disturbances in the coronary microcirculation have also been put forth as a potential mechanism for the development of rTTC [22]. Regarding TTC's prevalence, 90% of patients with TTC are postmenopausal women; hence, estrogen insufficiency has been suggested as a possible underlying cause [15]. Heart microvascular function is reduced by estrogen discontinuation. In oophorectomized rats, estrogen replenishment reduced the inhibitory effects of high epinephrine-induced cardiac contraction. This is hypothesized to be caused by a decrease in the plasma concentration of catecholamines and an increase in the B2 adrenoceptor signaling pathway. The estrogen in cardiac myocytes can control calcium uptake and affect cardiac contractility [14]. Less pulmonary edema, dyspnea, and cardiogenic shock may be seen in rTTC patients than in typical TTC patients [18]. This observation can be attributed to variances in the regional wall motion abnormality's location in the two cases, which resulted in different hemodynamic alterations and clinical characteristics. Apical ballooning, hypo-, a-, or dyskinesia of mid-apical myocardial segments is typical and sometimes associated with hypokinetic mid-segments. The combination of apical and mid segments represents the significant myocardial ischemic and dysfunctional area. That is why the typical TTC is more frequently associated with acute heart failure and pulmonary edema than the basal phenotype [23]. rTTC involves more cardiac tissue than traditional TTC. When compared to individuals with apical or midventricular TTC, patients with rTTC had higher levels of cardiac markers, including troponin-I and creatine kinase M (muscle type) or B (brain type) [24]. The degree of cardiac tissue damage in each class can account for this.

## Conclusions

rTTC SC is a rare variant of SC that presents with basal ballooning instead of apical ballooning seen in classic SC. It usually affects younger ages than the classic TTC. Despite the clinical presentation of rTTC usually being less severe than the classic TTC, the cardiac biomarkers levels are higher in rTTC due to the different myocardial segments affected. rTTC can happen in critically ill patients in the ICU secondary to severe sepsis and could contribute to hemodynamic worsening, affecting the final clinical outcomes.

## Appendices

Author contributions:

Design of the work: Muhammad Ghallab and Ibrahim Mohamed. Case writing: Muhammad Haseeb ul Rasool and Salma Abdelmoteleb. Data collection and interpretation: Allison foster, Zakaria Alagha, and Ashraf Sliem. Discussion and literature review: Ibrahim Mohamed, Salma Abdelmoteleb, and Zakaria Alagha. Drafting: Md Ripon Ahammed and Nicole C.Noff. Revision and approval of the article: Giovina Collura. Muhammad Ghallab is the article guarantor.
